# *Blastocystis sp*. in Irritable Bowel Syndrome (IBS) - Detection in Stool Aspirates during Colonoscopy

**DOI:** 10.1371/journal.pone.0121173

**Published:** 2015-09-16

**Authors:** Nanthiney Devi Ragavan, Suresh Kumar, Tan Tian Chye, Sanjiv Mahadeva, Ho Shiaw-Hooi

**Affiliations:** 1 Department of Parasitology, Faculty of Medicine, University of Malaya, 50603, Kuala Lumpur, Malaysia; 2 Department of Medicine, Faculty of Medicine, University of Malaya, 50603, Kuala Lumpur, Malaysia; Queen Mary Hospital, HONG KONG

## Abstract

*Blastocystis* is one of the most common gut parasites found in the intestinal tract of humans and animals. Its' association with IBS is controversial, possibly as a result of irregular shedding of parasites in stool and variation in stool detection. We aimed to screen for *Blastocystis* in colonic stool aspirate samples in adult patients with and without IBS undergoing colonoscopy for various indications and measure the interleukin levels (IL-8, IL-3 and IL-5). In addition to standard stool culture techniques, polymerase chain reaction (PCR) techniques were employed to detect and subtype *Blastocystis*. All the serum samples collected were subjected for ELISA studies to measure the interleukin levels (IL-8, IL-3 and IL-5). Among 109 (IBS n = 35 and non-IBS n = 74) adults, direct stool examination and culture of colonic aspirates were initially negative for *Blastocystis*. However, PCR analysis detected *Blastocystis* in 6 (17%) IBS and 4 (5.5%) non-IBS patients. In the six positive IBS patients by PCR method, subtype 3 was shown to be the most predominant (3/6: 50%) followed by subtype 4 (2/6; 33.3%) and subtype 5 (1/6; 16.6%). IL-8 levels were significantly elevated in the IBS Blasto group and IBS group (p<0.05) compared to non-IBS and non-IBS Blasto group. The level of IL-3 in were seen to be significantly higher in than IBS Blasto group and IBS group (p<0.05) compared to non-IBS. Meanwhile, the IL-5 levels were significantly higher in IBS Blasto group (p<0.05) compared to non-IBS and non-IBS Blasto group. This study implicates that detecting *Blastosystis* by PCR method using colonic aspirate samples during colonoscopy, suggests that this may be a better method for sample collection due to the parasite’s irregular shedding in *Blastocystis*-infected stools. Patients with IBS infected with parasite showed an increase in the interleukin levels demonstrate that *Blastocystis* does have an effect in the immune system.

## Introduction

Irritable bowel syndrome (IBS) is a chronic, functional gastrointestinal (GI) disorder which is common among adults in the West with a prevalence of 5–10% in Asia [1]. IBS is recognized as one of the commonest causes of consultation in gastroenterology clinics worldwide, resulting in a major economic burden for healthcare. The actual cause of IBS is still questionable, but major mechanisms are thought to include psychosocial factors, altered motility and hypersensitivity of the GI tract. Irritable bowel syndrome was defined based on the Rome III criteria [2] whereby gastrointestinal symptoms varied from diarrhoea, excessive intestinal gas, abdominal pain and bloating. Recent discovery of persistent, subtle inflammatory changes in adults with post-infectious IBS (i.e. IBS symptoms developing after an acute enteric infection) have alluded to an additional mechanism of intestinal infection mediated-inflammation [3]. Pathogens such as bacteria and parasites have also been a factor in contributing to the symptoms of IBS. Thus far, studies to determine the role of gut bacteria [4] and the type of gut microflora [5] seen in the intestine of IBS patients have been carried out.

*Blastocystis* is one the most common gut parasites found in the intestinal tract of humans and animals [6]. The organism exists in various morphological forms such as vacuolar, granular, amoeboid, cyst, avacuolar and multi-vacuolar forms and is transmitted through the fecal-oral route [7]. Both IBS and *Blastocystis* infected patients share common symptoms such as abdominal pain, diarrhea, constipation, cramps, nausea and fatigue [8]. The usual mode of diagnosis of *Blastocystis* in stool samples is via direct microscopy, *in vitro* culture technique and by polymerase chain reaction (PCR) method. A consensus terminology for subtyping *Blastocystis* was proposed by Stensvold et al (2007) and was categorized into nine subtypes which is found in human (ST1-ST9) [9].

Pathogenicity of *Blastocystis* remains disputable. A recent finding on the differing phenotypic and genotypic characteristics of this parasite isolated from asymptomatic and symptomatic patients [7] adds to the confusion. Various studies have implicated that genotypes of *Blastocystis* can clout the parasite’s pathogenicity particularly subtype 3 where in Malaysia [10], Singapore [11] and USA [12] evidence for its pathogenicity was clearly demonstrated. The rise of oxidative damage and proinflammatory cytokines by *Blastocystis* infection in animal models had been reported in our laboratory [13, 14]. Apart from that, solubilized antigen from *Blastocystis* (Blato-Ag) has the ability to downregulate PBMC while facilitates the growth of human colorectal cells [15]. A comparison study done in our laboratory demonstrate that Blasto-Ag from symptomatic isolates triggers a higher proliferation rate of colorectal cancer cells compared to Blasto-Ag from asymptomatic patient [16]. Blasto-Ag of ST3 revealed to be the most dominant subtype as to five other subtypes in triggering a higher proliferation rate in colorectal cancer cells [17]. Hence, *Blastocystis* does play an important role in contributing to the gastrointestinal symptoms. However, detecting this parasite in stool samples and cultures had been a challenge due to the irregular shedding of this parasite [18]. IBS patient choose colonoscopy as their last option when medication prescribed fails. Colonoscopy, whilst not indicated in all patients with IBS, is occasionally utilized to exclude organic causes of symptoms. Stool samples collected directly during colonoscopy (via aspiration) may circumvent many of the limitations of standard stool collection techniques for *Blastocystis* that we have described. In the present study, we attempted to assess the presence of *Blastocystis* among consecutive adult Malaysian patients undergoing colonoscopy. The prevalence of *Blastocystis* was compared between patients with and without IBS, according to Rome criteria III. *Blastocystis* does trigger the immune system of the patients and many studies had been done to study the pathogenicity of this parasite. In this study, the interleukin level such as Interleukin 8, 3 and 5 were measured to compare the interleukin levels between non-IBS patient, IBS patient, non-IBS patient and IBS patients both infected with *Blastocystis* respectively.

## Results

123 patients were screened during the study period and 109 (88.6%) patients agreed to participate in the study. Among the 109 patients, 35 (32.1%) and 74 (67.90%) were IBS and non-IBS respectively (Table 1). All stool aspirate samples were initially found to be negative for *Blastocystis* and all other parasites when examined by direct microscopy, formal ether concentration techniques as well as the *in vitro* culture method. Fresh stool samples were collected from all 109 patients 2 weeks after performing colonoscopy. Both the culture method and formal ether technique was negative for *Blastocystis* for all the 109 stool samples.

**Table 1:** Percentage of *Blastocystis* infection detected.

Group	Methods			
	Direct microscopy	In vitro cultivation	FECT	PCR
IBS (n = 35)	negative	negative	negative	6 (17.1%)*
non-IBS (n = 74)	negative	negative	negative	4 (5.5%)

*p < 0.05 is the comparison done between IBS and non-IBS patients.

**Fig 1:**
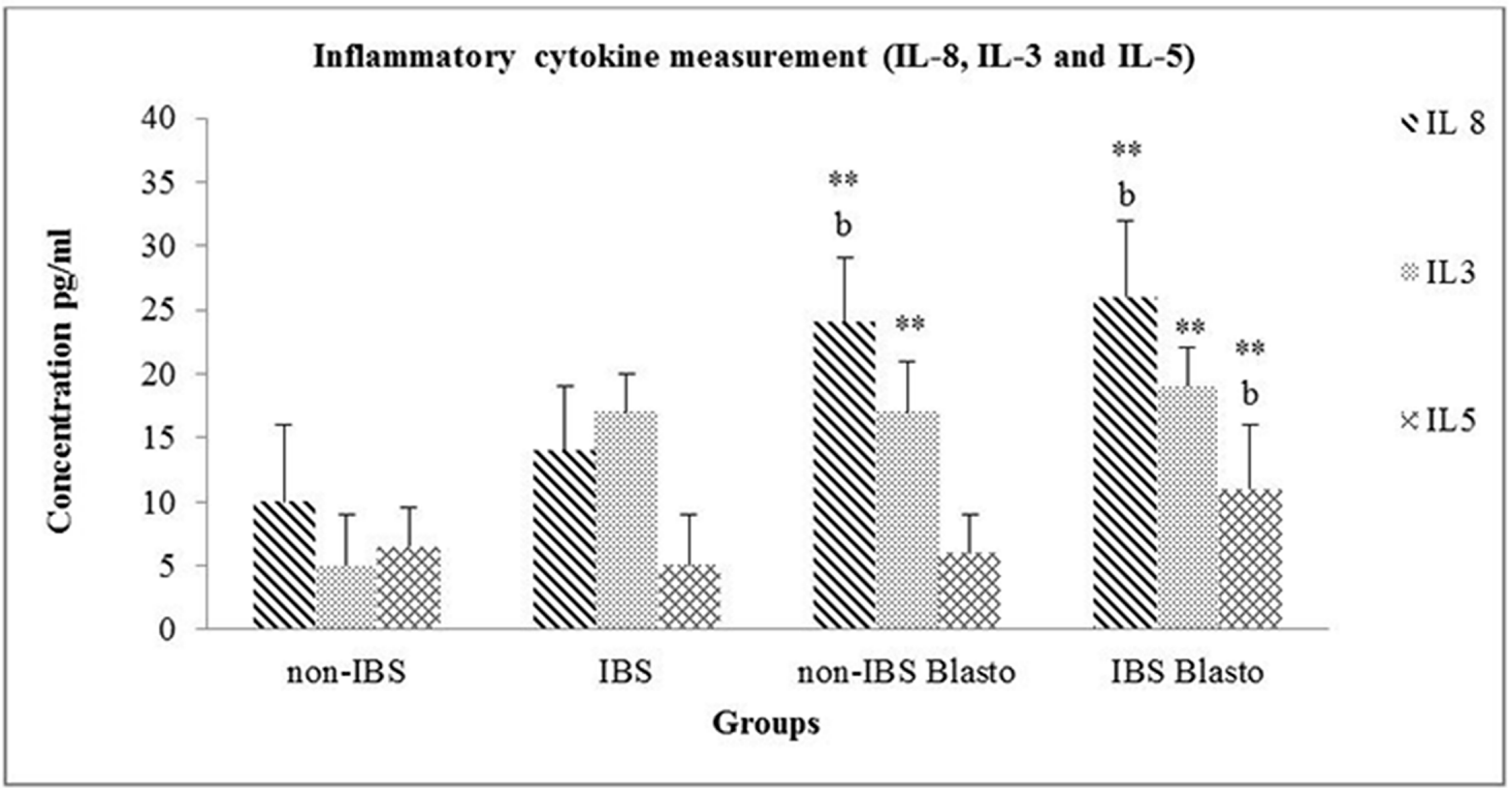
Levels of serum for IL 8, IL 3 and IL 5. Data is given in mean ± SD. **P < 0.05 is the comparison done against non-IBS group. ^b^P < 0.05 is the comparison done against IBS group.

However, when a PCR amplification method was utilised, we were able to identify *Blastocystis* in 6 (17.1%) IBS and 4 (5.5%) non-IBS patients (p = 0.047) (Table 1). Subtyping of *Blastocystis* were as follows: IBS (subtype 3; n = 3, subtype 4; n = 2 and subtype 5 n = 1) and non-IBS (subtype 2 n = 1, subtype 3 n = 2, subtype and subtype 5 n = 1). In this study, non-IBS are normal and healthy individuals, meanwhile non-IBS *Blastocystis* are asymptomatic individuals infected with *Blastocystis* and IBS *Blastocystis* group are *Blastocystis* infected IBS patients. Fig 1 demonstrate the comparison of serum cytokines IL-3, IL-5 and IL-8 between non-IBS, IBS, non-IBS *Blastocystis* and IBS *Blastocystis* groups. IL-8 levels were significantly elevated in the IBS Blasto group and IBS group (p<0.05) compared to non-IBS and non-IBS Blasto group. The level of IL-3 in were only seen to be significantly higher in IBS Blasto group and IBS group (p<0.05) compared to non-IBS. Meanwhile, the IL-5 levels were significantly higher in IBS Blasto group (p<0.05) compared to non-IBS and non-IBS Blasto group.

## Discussion

The pathogenicity of *Blastocystis* is still disputable as adults with an acute infection are known to develop gastro-intestinal symptoms similar to IBS, usually following travel to an endemic geographical location. However, its' association with IBS, a chronic GI disorder, is less certain. The irregular shedding of *Blastocystis* in stools as well as the intermittent shedding of other protozoa such *G*. *intestinalis* and *D*. *fragilis* does imply that single stool examination has a low sensitivity for detecting [18]. In patients with *E*. *histolytica*, up to four to nine separate fecal examinations were required to make the diagnosis. With additional stool examinations, the diagnostic yield has been shown to increase by 22.7% for *E*. *histolytica*, 11.3% for *G*. *intestinalis* and 31.1% for *D*. *fragilis* [19].

A prevalence study done in Thailand shows that 13.6% of IBS patients infected with *Blastocystis* compared to 20% of normal patients found to be not statistically significant [20]. Another study done by Surangsrirat et al (2010) on the IBS patients infected with *Blastocystis* found to be not statistically significant which 10.0% and 16.7% were positive for in both control and IBS patients respectively [21]. However, in our current studies, we found that the results were statistically significant, 17% (6/35) of IBS patients were infected with *Blastocystis* compared to 5.5% (4/74) control patients. This present study is the first study in Southeast Asia to show that the results of associating *Blastocystis* to IBS were statistically significant. However, the prevalence of *Blastocystis* among IBS patients in our study was somewhat lower than previous published report. This may have been due to patient selection in our study, as all patients were undergoing colonoscopy, which is not commonly indicated in patients with IBS. Our present study also showed that PCR method used in colonic stool aspirate samples was more sensitive than direct microscopy, *in vitro* culture method and trichrome stain. Purgatives and rapid colonic transit prior to colonoscopy may have flushed all the parasites out providing an initial negative result for direct microscopy, *in vitro culture* technique and trichrome staining technique. Traces of DNA of *Blastocystis* were able to be detected by the PCR technique demonstrating that PCR could be a better method in detecting this parasite in IBS patients who were undergoing colonoscopy. This study suggest that IBS patients who are undergoing colonoscopy should be screened for this parasite by using PCR method in order for them to eradicate this parasite immediately if at all they are found to be positive for *Blastocyostis*. This present study support the earlier research done by Vinoth et al (2014) suggesting that colonic washout can be an advantage to detect the parasite as the samples are collected directly from the washout during colonoscopy due to the irregular shedding of *Blastocystis* [17]. However, the study done was confined on the prevalence and detecting techniques only.

The pathogenicity of *Blastocystis* is still debatable due to its existence in both asymptomatic individuals and symptomatic patients [22, 23]. Hence, in this study, it is pertinent to look into the immune system of the non-IBS patients, IBS patients and both normal and IBS patients infected with *Blastocystis*.

IL-8, a chemoattractant for neutrophils and macrophages also is known to act as pro-inflammatory mediator in cellular responses [24, 25] as seen previously when vaginal tract exposed to *T*.*vaginalis* showed stimulated IL-8 indicating chronic inflammation [24]. According to Kollmar (2006), the expression of IL-8 is important in tumour progression via the alteration of the immune system and the regulation of tumour cell growth [26]. The levels of interleukin were higher in IBS than non-IBS group implicating *Blastocystis* infection to have an effect on inflammatory cytokines released by the cells. However, the level of interleukin varies between non-IBS Blasto and IBS Blasto group. A recent study done by Chandramathi et al, (2012) reported that *Blastocystis* in patients possibly immunosuppressed due to chemotherapy could tend to become opportunistic and exploit the advantageous situation to multiply more causing symptoms to exacerbate [27]. Nanthiney et al (2014) demonstrated that phenotypic characteristics of *Blastocystis* isolated from IBS patients showed various unique and different phenotypic characteristics compared to the same parasite isolated from symptomatic and asymptomatic patients. This was attributed to the adaptation of the parasite to the fluctuations of the gut environments especially in IBS patients [28].

We conclude that PCR examination of stool aspirates during colonoscopy is a useful method of identifying the presence of *Blastocystis* in adult patients with IBS. Whilst colonoscopy remains invasive and clearly not indicated in all symptomatic adults, it may be considered for patients negative for stool culture but with a high suspicion of *Blastocystis* based on its characteristics symptoms. IBS patients with *Blastocystis* showing elevated levels of interleukin demonstrate that *Blastocystis* does have an effect in the immune system.

## Materials and Methods

### Sample Collection

A hospitalized-based cross sectional between May 2010 and May 2011 whereby adult patients attending this institution’s weekly colonoscopy list for an index examination for various indications were invited to participate in the study. A single investigator interviewed all patients prior to colonoscopy and irritable bowel syndrome was defined according to the Rome III criteria [2] together with a normal or insignificant colonoscopy finding. Patients with a recent use of antibiotics, particularly Metronidazole, were excluded. A standardized bowel preparation regime consisting of bisacodyl and low-residue diet for two days followed by a 2-liter polyethylene glycol and electrolyte lavage solution (PEG-ELS) was used for all patients undergoing colonoscopy at this institution. Colonoscopy was performed using standard video-endoscopes with variable stiffness (CF 160AL, Olympus, Tokyo, Japan). All patients received a combination of Midazolam 2.5 mg to 5 mg and Pethidine 25 mg to 50 mg as sedation prior to colonoscopy. The protocol for this study was approved by our local institutional ethics committee.

### Stool Processing

Stool aspirate samples were collected in 1500cc CRD Liners following direct aspiration during colonoscopy from the IBS and non-IBS patients. The samples were then transported to the Department of Parasitology and stool aspirate samples were then spun using 50ml Falcon tube at 3000rpm for 10 minutes and sediment assessed for the parasite using direct microscopy as well as introduced 50mg of the sediment were then cultured in 3ml Jones medium supplemented with 10% horse serum and kept in incubator at 37°C. The cultures were screened for *Blastocystis* after 24, 48 and 72 hours using light microscopy. Formal ether concentration techniques (FECT) were also done to screen for the presence of other intestinal parasites. Various staining techniques were carried out such as Ziehl- Neelsen, modified trichrome and trichrome staining methods to detect for *Cryptosporidium*, microsporidia and other parasites as well. Samples of colonic lavage samples were kept at 4°C for deoxyribonucleic acid (DNA) extraction using QIAamp DNA stool mini kit (Qiagen, Hilden, Germany).

### Subtyping of *Blastocystis*

The genomic DNA of *Blastocystis* for all colonic washout collected were extracted using QIAamp DNA stool mini kit (Qiagen, Hilden, Germany) based on the manufacturers’ protocol. All the samples were subjected to sequence tagged site (STS) primer-polymerase chain reaction (PCR) using the seven sets of primers previously described by Yoshikawa et al. (2004b) [29]. Two to five microliters of DNA preparations were used to amplify the genomic sequences in a 20μl reaction reaction containing 1x PCR buffer (Fermentas, USA). PCR condition consisted of 1 cycle of initial denaturing at 95°C for 5 minutes, followed by 40 cycles of denaturing at 95°C for 1 minute, annealing at 56.3°C for 1 minute 30 seconds and extending at 72°C for 1 minute, and an additional cycle of elongation at 72°C for 10 minute (Thermocycler Biorad). The amplified products were then electrophoresed in 1.5% agarose gels (Promega, USA) in Tris–borate-EDTA buffer. Gels were stained with ethidium bromide and photographed using an ultraviolet gel documentation system (Uvitec, United Kingdom). PCR amplification for each primer pair were done in triplicate.

### Blood Sample Collection

Blood samples were collected from all the patients who came to the colonoscopy unit. 3cc blood samples were collected in plain EDTA tubes. Blood was allowed to coagulate and spin at 2000rpm for 10 minutes to obtain the serum samples. The serum samples were kept at -20°C until inflammatory cytokine measurements was done.

### Inflammatory Cytokines

Human IL-8, IL-3 and IL-5 ELISA (Enzyme-Linked Immunosorbent Assay) purchased from Bio-Rad (Raybio) were used in this study. This assay uses an antibody which is specific to IL-8, IL-3 and IL-5 coated on a 96-well plate. All reagents, samples and standards were prepared as instructed. 100μl of sample were added into each well and incubated for 2.5 hours at room temperature. The specific interleukins (IL-8, IL-3 and IL-5) which present in the serum samples will bind to the wells by the immobilized antibody. The wells are washed and 100μl of Biotin antibody were added and incubated again for one hour at room temperature. After washing away unbound biotinylated antibody, 100μl of HRP conjugated streptavidin is added into the wells. The wells are again washed and 100μl TMB substrate solution is added to the wells and colour develops in proportion to the amount of respective interleukin bind. The Stop Solution develops the colour from blue to yellow, and the intensity of the colour is measured at 450 nm.

### Statistics

Double data entry was performed using Microsoft Excel 2010 and statistical analyses conducted with IBM SPSS version 21. For purposes of analysis, patients' diagnoses were classified as IBS, non-IBS, IBS Blasto and non-IBS Blasto. The latter consisted of any clinically significant colonoscopy findings. Chi-square analysis test was used to examine the differences in prevalence of *Blastocystis* in IBS and non-IBS patients. The inflammatory cytokines measurement was analyzed using Student T test. Statistical significance was defined as a p value of <0.05.
